# Online Measurement for Parameter Discovery in Fused Filament Fabrication

**DOI:** 10.1007/s40192-024-00350-w

**Published:** 2024-04-03

**Authors:** Jake Robert Read, Jonathan E. Seppala, Filippos Tourlomousis, James A. Warren, Nicole Bakker, Neil Gershenfeld

**Affiliations:** 1 Center for Bits and Atoms, Massachusetts Institute of Technology, Cambridge, MA 02143 USA; 2 Materials Science and Engineering Division, National Institute of Standards and Technology, Gaithersburg, MD 20899 USA; 3 Superlabs, National Center for Scientific Research Demokritos, Athens, Greece; 4 Media Lab, Massachusetts Institute of Technology, Cambridge, MA 02143 USA

**Keywords:** Fused filament fabrication, Parameter selection, Parameter transfer, Process tuning, Additive manufacturing

## Abstract

To describe a new method for the automatic generation of process parameters for fused filament fabrication (FFF) across varying machines and materials. We use an instrumented extruder to fit a function that maps nozzle pressures across varying flow rates and temperatures for a given machine and material configuration. We then develop a method to extract real parameters for flow rate and temperature using relative pressures and temperature offsets. Our method allows us to successfully find process parameters, using one set of input parameters, across all of the machine and material configurations that we tested, even in materials that we had never printed before. Rather than using direct parameters in FFF printing, which is time-consuming to tune and modify, it is possible to deploy machine-generated data that captures the fundamental phenomenology of FFF to automatically select parameters.

## Introduction

Fused filament fabrication (FFF) [1] is a rapid prototyping process, where tracks of molten polymers are extruded line-by-line and layer-by-layer through a heated nozzle in order to build a part. The process continues to rise in popularity due to its low cost and simple nature, and is especially prevalent among open source machine builders, where a proliferation of new machine designs and material options is continually emerging.

FFF printing requires that part geometries be transformed into machine instructions in a process called *slicing*, to do so we use software packages aptly named *“slicers”*. In order to print successfully, slicers must be configured such that the instructions they generate work with the particular machine and material being used downstream. For example, two of the most relevant configuration parameters that must be selected are the nozzle temperature and the material flow rate; the first is set directly and the second is set indirectly as a function of track width, height, and speed. These parameters relate to the materials’ properties as well as to the particulars of the machine: nozzle diameter is of course one major factor affecting flow rate, as is the overall thermodynamics of the nozzle (i.e., melt zone length and shape) and the extruder’s ability to produce pressure.

Instead of inferring optimal parameters from state-of-the-art models, most slicers deploy process parameter sets that are hand-tuned via extensive trial and error. Not only this is time-consuming, it is also non-transferable across machines or materials: one parameter set is unique to one complete FFF configuration, meaning a machine (hotend design and nozzle diameter) and a material. This leads to wasted time, material, and sub-optimal prints, and especially presents a challenge to those among us who build or modify their machines to perform beyond where most heuristic sets have been refined, or who use novel materials that are recycled [2, 3], derived from biological origins [4], or have advanced properties including cell-free and cell-laden bioinks [5] and conductivity for additive electronics production [6].

The development of machines that can forgo this hand-tuning process may speed the development of new FFF printers and the adoption of new, renewable and recycled FFF materials. We try to do so in this paper. However, rather than backing into a complex modeling exercise, we develop a workflow that deploys a simple function fit with an online data gathering routine to automatically select process parameters using an instrumented extruder that extends work from *Coogan and Kazmer* [7]. The workflow replaces roughly half of the hand-tuned parameters in state-of-the-art slicers with one dataset (generated with the matching machine and material within tens of minutes and tens of grams of filament) and one additional input parameter that specifies temperature and flow rate in relative terms.

We found that our method can consistently pick viable print parameters for known and unknown materials when we used it to print a series of benchmark models using machine configurations that we had not tested previously. We were also able to do this using the same input parameter across all configurations. We hope that this method will be especially relevant to the emergence of advanced and sustainable material blends, whose adoption is hampered by users’ not having reliable access to viable print parameters.

In this paper we provide some background on the FFF process in “The FFF Process, Extruder, and Limitations,” and also an overview of how FFF process parameters are articulated in state-of-the-art slicers in “Typical FFF Parameter Sets.” In our methods section “Methods” we provide detail on our instrumented extruder (“Instrumented FFF Extruder”) and data gathering routine (“Data Gathering and Normalization”), as well as the shape of our function fit (“Function Fitting”) that maps pressure as a function of flow rate and nozzle temperature set point $P = f(Q, T)$. In “Extracting Real Parameters from Function Fits using Input Parameters,” we explore the connection between our function fits for poly lactic acid (PLA) and acrylonitrile butadiene styrene (ABS) and heuristically developed parameter sets for the same, and show how we selected a single input parameter for any configuration. We summarize how the system is deployed in “System Summary.” Finally in our “Results” section we deploy our workflow on a litany of materials and on two machine configurations. Finally, we discuss limitations and future work in “Limitations and Future Work” and conclude the paper in “Conclusion.”

### Related Work

Although some slicers can directly transmit low-level instructions to machines [8], and other tools omit the slicer entirely such as *FullControl GCode Designer*^1^ in which users create print paths with Microsoft Excel [9] and *p5.fab* for direct control over FFF printing parameters through creative coding [10], only one that we found can read data or configurations directly from an FFF machine; all others are configured in a feed-forward manner. The *ORNL Slicer 2.0* developed by Oak Ridge National Laboratory is based on an on-demand process that gathers sensor information at each layer and provides feedback to the slicer, before generating partial G-code for the next layer. Sensors are used in the form of thermal cameras and laser profilometers [11]. Other instrumented printers that measure quality variables with sensors include: [12] and [13]. Kumar et al developed a low-cost multi-sensor strategy for error detection during FFF printing, and used sensors for measuring vibration, current, and sound [14].

Perhaps the closest aligned work to our own are two vision-based methods [15] and [16]. While these two methods are more effective parameter fine-tuners, they require an initial set of printing conditions that produce viable output, whereas ours does not. They also both require more input data than does our method. That said, their end results are of a higher overall quality than ours, meaning that a combination of our method (to set initial conditions) and vision-based fine tuning is a viable path toward optimal printing.

The physics of FFF printing are well understood in the literature [17, 18] and it is likely possible to develop full-scale models of the FFF process that could relate material models directly to machine models in order to pick optimal slicer configurations. However, to our knowledge no-one has made substantial effort to apply these models to automatically select parameters for FFF machines, although much work has been done to evaluate the effects of parameter selection on the quality of printed outputs [19–24]. The focus in this work is on how to rapidly select operating parameters from a short, online rheological experiment.

### The FFF Process, Extruder, and Limitations

**Fig. 1:**
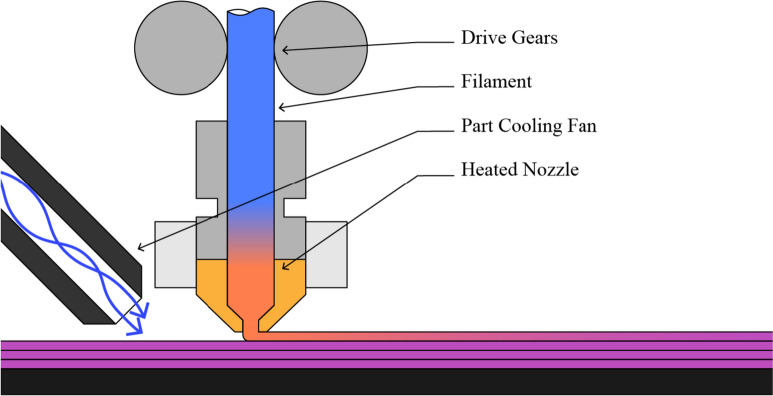
This figure shows a diagrammatic example of a typical FFF extruder, where a cylindrical filament is pushed, using hobbed drive gears, through a heated nozzle to precisely lay tracks on a moving bed

FFF is simple in principle but becomes complex when examined in closer detail. We provide a diagrammatic representation of the basics in Fig. 1. In a coarse view, FFF machines push a thermoplastic filament into a heated cylinder using hobbed drive gears. As the filament travels through this *hotend* it melts, and is extruded out of a small diameter nozzle (e.g., 0.4mm). The molten extrudate is deposited in tracks, which are composed into layers and subsequently complete parts, by moving the nozzle very precisely as this extrusion is going on.

Inside the nozzle and melt zone, classical rheological models can be easily applied [25]. Indeed, [7] uses an instrumented extruder similar to the one in this work to fit data to these rheological models, showing that much of the FFF process can be modeled as such. These models can tell us how much pressure needs to be generated inside of a nozzle of a given shape, with a given polymer, in order to achieve a given flow rate.

However, real-time operation of an FFF machine is often much more dynamic than this, especially because flow rates are constantly changing; it is important to remember that even though feed rates are set at constant velocities, machine controllers are continuously changing actual velocities as they limit acceleration into and out of corners [26]. This means that models appropriate for steady-state rheology may not map well into real FFF operation.

At maximum flow rates, system limits are almost entirely thermodynamic [27]. Acknowledgment of this insight is evident in the FFF community’s recent deployment of nozzles like the *Bondtech CHT* [28] and the *E3D Revo High-Flow* [29] that both increase nozzle to filament surface area (to improve conduction) in order to increase flow.

The nozzle is only one component of the complete dynamics of the FFF process. Also important to consider is the mechanical limit to nozzle pressure generation [30]. FFF extruders typically use hobbed shafts that are preloaded into the filament in order to drive material into the nozzle. Filaments eventually shear under the stresses exerted on them by these hobbed shafts, meaning that only a limited amount of pressure can be supplied to the nozzle. This limit is acknowledged in the design of the *Prusa Nextruder,* which increases the extruder’s ability to generate pressure by increasing the number of sites at which the extruder’s hobbed gear is engaged with the filament.

Further, complexity in FFF can be found outside of the extrusion process itself. As we will see in this work, flow can always be increased by increasing nozzle temperature, but over-heating filament in the nozzle can lead to slumping of the printed part. This is simple to understand: once printed, the filament is unconstrained and if it is too molten it will not hold its deposited shape. To compound this, the filament is typically resting on a previous layer of filament, and so prints need to be strong enough, as they are being printed, to remain self-supporting. This phenomenology has led to the inclusion of ‘part cooling fans’ in most extruder designs that allow nozzle temperatures to remain large while quickly cooling filament on exit to avoid slumping.

Slumping would be perhaps the most complex aspect of FFF to capture accurately; a prospective modeler would need not only to understand the nozzle and extruder, but also the part geometry itself, the machine’s complete set of motion dynamics (to estimate real layer times), and information about the part’s cooling rate, the materials’ own thermodynamic properties, and perhaps even expectations about the ambient temperature and airflow around and within printer.

### Typical FFF Parameter Sets

Rather than try to model all of these process intricacies (and additionally try to articulate what “optimal” configurations might be), state-of-the-art slicers simply use a large number of user-specified feed-forward settings (a *configuration*) in order to develop their outputs. These configurations are hand-tuned via trial and error and are specific to a complete *machine, material* set; whenever a nozzle diameter, extruder design, *or* material is changed, a new configuration must be developed or adapted.

**Table 1:** To understand how FFF machine instructions are generated in practice, we include here, a table of settings from *PrusaSlicer* that affect flow rates and temperatures directly

Settings section	Setting	Units	Typical$^1$
Filament settings/filament	Temperature	$^{\circ }$C	215
Filament settings/advanced	Max volumetric speed	mm$^3$/s	15
Print settings/layers and perimeters	Layer height	mm	0.2
Print settings/speed	Perimeters	mm/s	45
	Small perimeters	mm/s	25
	External perimeters	mm/s	25
	Infill	mm/s	80
	Solid infill	mm/s	80
	Top solid Infill	mm/s	40
	Supports	mm/s	50
	Supports interface	%	80
	Bridges	mm/s	25
	Gap fill	mm/s	40
Print settings/advanced/width	Default extrusion	mm	0.45
	First layer	mm	0.42
	Perimeters	mm	0.42
	External perimeters	mm	0.42
	Infill	mm	0.42
	Solid infill	mm	0.42
	Top solid infill	mm	0.4

$^1$These reference values are included from a configuration file for “Generic PLA” extruded through a 0.4mm diameter nozzle with an E3D V6 hotend. This closely matches the reference configuration of our instrumented extruder

**Table 2:** Volumetric flow rates are not directly exposed in slicer configurations

Track type	Height mm	Width mm	Rate mm/s	Flow rate mm$^3$/s
**0.2mm “Quality”**				
Perimeters	0.2	0.42	45	3.78
External Perimeters	0.2	0.42	25	2.10
Infill	0.2	0.42	80	6.72
Supports	0.2	0.45	50	4.50
**0.2mm “Speed”**				
Perimeters	0.2	0.42	60	5.04
External Perimeters	0.2	0.42	25	2.10
Infill	0.2	0.42	200	**16**.**8**
Supports	0.2	0.45	50	4.50

Here, we use indirect settings from Table 1 to calculate some resulting flow rates. Track types that are configured to exceed maximal flow rates are bolded

Many settings are geometric in nature (layer height, infill density, infill patterns, and shell thicknesses), and we consider these settings to be outside the scope of this paper. Here, we are primarily concerned with what we see as the two most important (and difficult to determine) parameters, which are print speeds (in terms of flow rates) and temperatures. In our survey of two popular slicers, some data from which is available in Table 1, nearly half of the total settings available in any given configuration were related to flow rate and temperatures (we present only these settings in the table), but the relations were all indirect. For example, flow rate appears directly only once, in the aptly named *Max Volumetric Speed* setting: elsewhere it is encoded indirectly by a combination of layer height, track width, and linear speeds. In Table 2, we calculate actual flow rates for a few different track types given typical values. Some of these flow rates exceed maximum flow rates (as specified elsewhere), we present those in bold. Nozzle temperatures at least are uncomplicated and direct, and are assigned per material.

Since a considerable number of hours have been spent by FFF community members tuning these values, we can assume that they contain some insight as to how FFF machines should be operated, even though the exact logic behind any given value is not explicitly clear. The first take-away from these parameters is that speed is often reduced when detail or precision is required (i.e., on *external* and *small perimeters*, and is maximized (toward apparent maximal volumetric flow rates) when it is not so important (i.e., during *infill*). However, flow rates and temperatures are not all that informs these values: lower speeds also imply higher quality of motion from a machine’s linear axes and dynamics.

## Methods

Given the development of instrumented extruders, we wanted to develop a method for print parameter selection that lay somewhere between the use of complete and complex models of the FFF process [31] (which seems daunting and messy), and state-of-the-art feed-forward (and blind) solutions. We also wanted our process to be deployable as an online solution; something users might run just ahead of any new print, or whenever they load new filament into a machine.

To do so, we developed a simple function fit that relates typical nozzle pressure to an operating temperature and flow rate $P = f(T, Q)$ that we can generate using data that only takes a few minutes to collect.

Then, using the function fit, we can extract real parameters using input parameters that describe temperatures $T_{rel}$ as offsets from initial flow conditions, and that select flow rates based on pressures $P_{sel}$ relative to the extruders’ maximum pressure.

Together, these methods combine into a workflow that we describe in “System Summary,” where novel materials can be loaded into our printer, a set of parameters can be automatically generated and loaded into a slicer software, and prints can be carried out.

To aid in other researchers’ reproduction and extension of this work, we have published a Git repository at https://gitlab.cba.mit.edu/jakeread/online-measurement-for-parameter-discovery-in-fff that includes mechanical designs, circuit designs, and source codes for the firmware, frameworks, and experiments discussed in this paper.

### Instrumented FFF Extruder

**Fig. 2:**
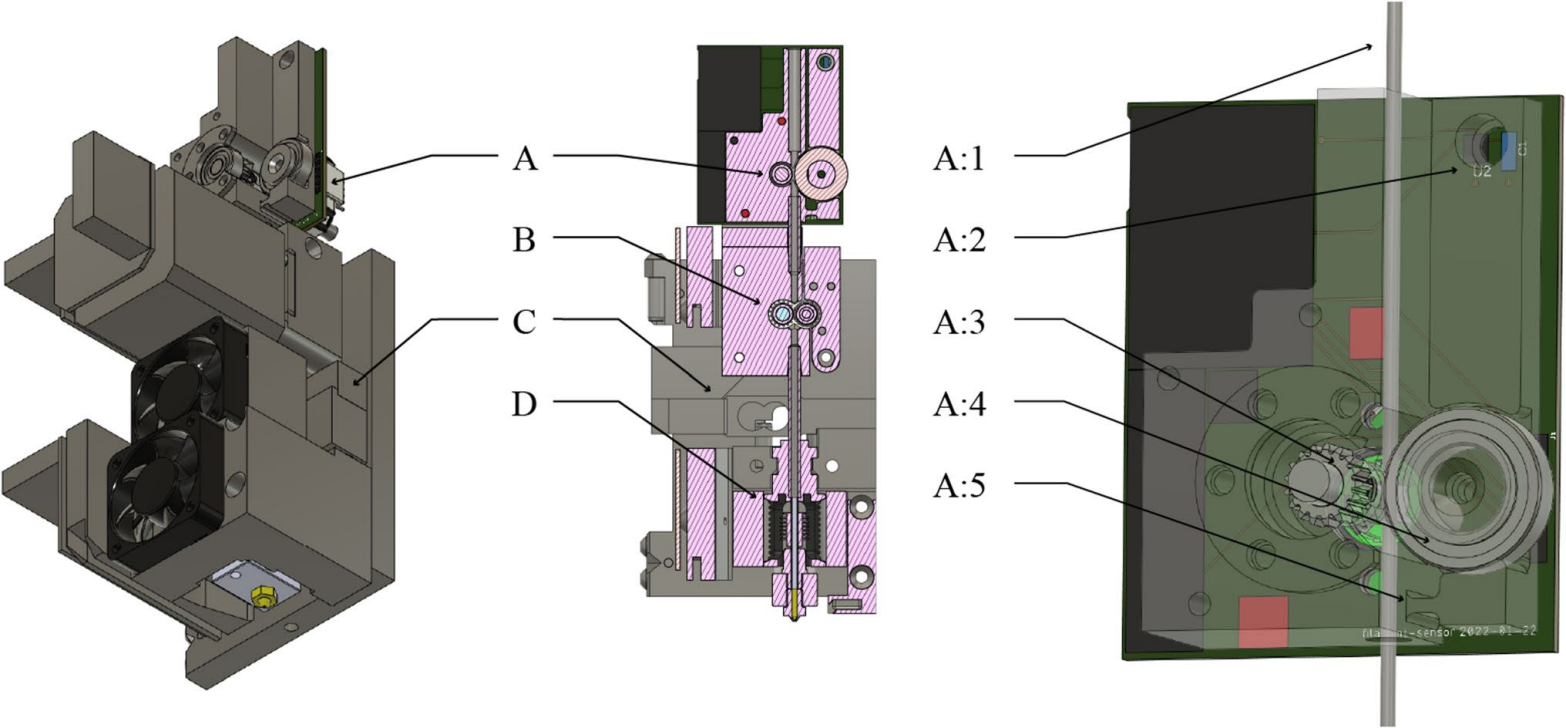
We designed and built an instrumented extruder for FFF 3D Printing. It is largely the same as most Commercial Off-the-Shelf (COTS) FFF extruders, using and E3D V6 hotend (D) and Bondtech drive gears (B). To this assembly we add a load cell (C) that sits in the middle of the structural loop between the drive gears and the hotend, meaning that it measures all of the force exerted by the drive gears onto the filament. We use this reading as a pressure analog. Additionally, we developed a filament sensor (A) that measures the real linear feed rate of the filament (A:1) using an idler gear attached to an encoder (A:3) preloaded by an idler roller (A:4). The roller is preloaded using a flexural hinge (A:5) and a lever arm. A hall-effect sensor (A:2) reads the displacement of this lever arm; these readings are calibrated and used to measure real filament diameter. More detailed figures and CAD models of these components are included in the repository referenced at the beginning of this section

Following work on in-line rheological monitoring [7], we designed and built an FFF extruder (shown in Fig. 2) that allows us to measure a nozzle pressure analog and to detect filament slip at the drive gears. We render the extruder here in Fig. 2.

While [7] used an in-line pressure transducer in their work, we avoid the costly and complex nozzle modification by instead measuring pressure indirectly. Our extruder mounts the hotend to the machine chassis via a load cell, meaning that any force exerted by the filament on the nozzle is measured in this load cell. This has the possible disadvantage of reading external forces as well (such as friction between the filament and the hotend tube’s sidewall), and forces exerted on the nozzle by (for example) existing tracks of filament, but we found the measurements useful regardless, as our work does not yet attempt to measure nozzle pressure during printing.

We additionally developed an instrument that measures the width and linear feed rate of the filament before it enters the nozzle, based on a design from [32]. This is also pictured in Fig. 2. It does so with two idler wheels, one of which is hobbed in the same manner as the extruder’s drive gears, the other of which is passive. The hobbed idler is fitted with a rotary encoder to sense linear feed rate of the filament and the other is attached to a swing-arm, whose displacement is analogous to changes in filament thickness. Together, these readings can tell us the real volumetric feed rate of filament into the extruder. In this work, we use this instrument solely to detect filament slip, i.e., cases where the extruder’s linear feed rate reads near zero but the drive motor is continuing to spin.

While our extruder is instrumented, its performance should be fundamentally similar to many other consumer FFF printers, since it uses the *E3D V6* hotend and *Bondtech* drive gears, which have emerged as pseudo-standards in low-cost printer designs. These are also the main components that contribute to extruder phenomenology, and are the same as those used in the machine that matches our reference heuristics from “Extracting Real Parameters from Function Fits using Input Parameters.”

### Data Gathering and Normalization

Each component of the hotend is fitted with a custom-designed circuit and local control logic. Devices are connected over a network to one another and to a systems coordinator, written in JavaScript that allows us to quickly write high-level routines for data collection [33]. More details on this system are available in the repository referenced in the beginning of this section. We used this system to develop a simple data gathering routine whose steps are enumerated below.

**Fig. 3:**
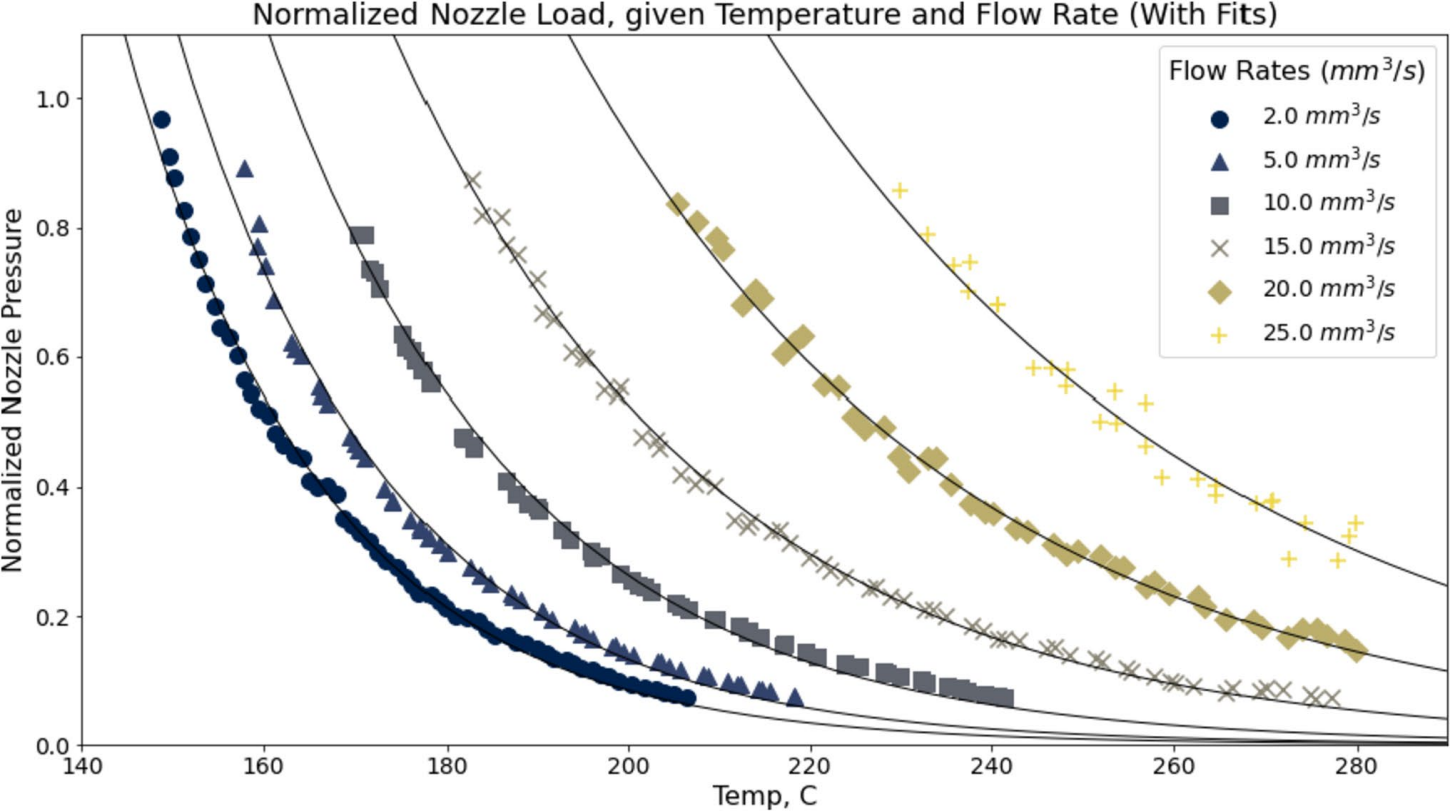
Here, we show cleaned data traces from samples taken across five flow rates 5$\hbox {mm}^{3}/s$ to 25 $\hbox {mm}^{3}/s$ for ABS through a 0.8mm nozzle on an E3D V6 hotend. During each trace, we set the hotend to near its maximum temperature of 290${^\circ \hbox {C}}$, begin flowing filament at the requested rate, and then simply turn the heating element off in the extruder. The resulting time-series gathers nozzle pressure (as a raw load cell reading, normalized from $0 \rightarrow 1$), across a decreasing range of temperatures (and increasing pressures) as the nozzle cools naturally. Each point here, is an individual data point. They are collected at 200m s intervals. At a certain point, the extruder is unable to drive filament at the operating pressure, and slip occurs. Our filament sensor detects this slip, and the experiment is terminated. This figure also includes traces from our preliminary fit, which fits the data against $P = a^{T + b}$ where *P* is normalized pressure and *T* is the nozzle temperature set point, as discussed in section “Fitting Individual Flow Rates.”

1. The hotend is heated to its maximum temperature, or to the upper bound of the desired dataset. In our case, this was 290${^\circ \hbox {C}}$.
2. The hotend is purged with 10mm of filament.
3. The extruder is set to extrude continuously at the desired flow rate.
4. The hotend is turned off and allowed to cool toward ambient temperature, while filament continues to be pushed into the hotend.
5. While filament is being extruded, we record a time-series of samples from the extruder’s load cell, filament sensor, and thermocouple at 200m s intervals.
6. We continuously use the filament sensor to estimate of the extruder’s real feed rate against the requested rate. This gives us a drive percentage, where 100% indicates zero slip. We terminate the run when this value drops below 75%, indicating major failure of the extruder to generate adequate force. We then store the dataset for later analysis.

This procedure results in a series of pressure vs temperature traces, each at a different flow rate. Figure 3 shows a series of these traces, each with a preliminary exponential fit, whose parameters are rendered in Table 3. Traces can take between 90 s and five minutes to complete, meaning that (depending on the fidelity desired) characterizing a new machine and material configuration takes between 10 and 30 min.

Data taken when the extruder is operating at relatively low nozzle pressures were quite noisy, and so we exclude data points whose pressure readings are in the bottom 15% of the maximal (final) pressure. We additionally exclude data in the top 10% of the pressure range, since points at or near extruder-gear slip are equally noisy.

Load values are normalized to span a simple $0 \rightarrow 1$ range, where 1 represents the maximum extrusion force obtainable from the system before inducing slip, i.e., we use $P = P_{reading} / P_{max}$ where $P_{reading}$ is the raw load cell reading (which we take to be linear, but do not calibrate) and $P_{max}$ is the largest reading taken with the given configuration.

We chose not to calibrate our load cell values because the additional operating complexity could be prohibitive in deployed systems, and because it introduces opportunity for user error. We also presumed that normalizing to the machine’s own maximal extrusion force would provide enough utility (allowing us to pick viable parameters); i.e., it is not necessary in this case to know the *real* pressures generated in the nozzle, only the *relative* pressures. We acknowledge that this limits our ability to compare data between two different machines, or to perform more advanced rheology on the data.

### Function Fitting

#### Fitting Individual Flow Rates

Once data are collected and cleaned, we do a preliminary function fit for each unique flow rate against a generic exponential function 1.

1 $$\begin{aligned} P = a^{T+b} \end{aligned}$$

where *P* is the expected normalized pressure at temperature $T (^{\circ }C)$ and *a*, *b* are parameters that we fit using the Levenberg-Marquardt algorithm as implemented in the *scipy* compute package [34]. A sample of these fits is rendered in Fig. 3 and Table 3.

The function fit our data well, and were encouraged to find $a \ and \ b$ parameters were somewhat interpretable; the *b* parameter maps nicely to the temperature where nozzle back pressure exceeds the extruder’s drive gear traction (i.e., where slippage begins to occur) and functions as an effective minimum temperature for the given flow rate. The *a* parameter then maps to the rate at which nozzle pressure drops off, at the given flow rate, as temperature is increased. For example with small flow rates *a* has a stronger exponent $(a \approx 0.95)$, meaning that pressures drop drastically as temperature increases, whereas large flow rates drop off less drastically $(a \approx 0.99)$.

**Table 3:** Here, we show fit parameters for data traces rendered in Fig. 3 that match data against $P = a^{T + b}$ where *P* is normalized pressure and *T* is the nozzle temperature set point, as discussed in section “Function Fitting”

Flow rate *Q* (mm$^3$/s)	*a*	*b*
5.0	0.953	−155
10.0	0.960	−167
15.0	0.970	−181
20.0	0.976	−201
25.0	0.980	−225

We suspect that these changes in *a* relate mostly to the thermodynamics of the melting filament. Recalling that our hardware only measures the hotend temperature at some point in the heat block (not the actual melt flow temperature) we can make some sense of this. At lower flow rates, any given section of filament spends more time in the hotend’s melt zone, meaning there is more time to complete the heat transfer. This correlates to smaller values of *a*, i.e., more pronounced decrease in pressure with respect to temperature; all of the temperature increase is realized in the melt flow. On the other hand, larger flow rates correspond to smaller drops in pressure with respect to temperature, since the filament does not have enough time in the melt zone to completely come up to the nozzle’s set point temperature. In section “Extracting Thermodynamic Models from Data Traces,” we discuss the possibility of extracting a thermodynamic model more directly, using the same data.

#### Fitting Entire Operating Spaces

**Fig. 4:**
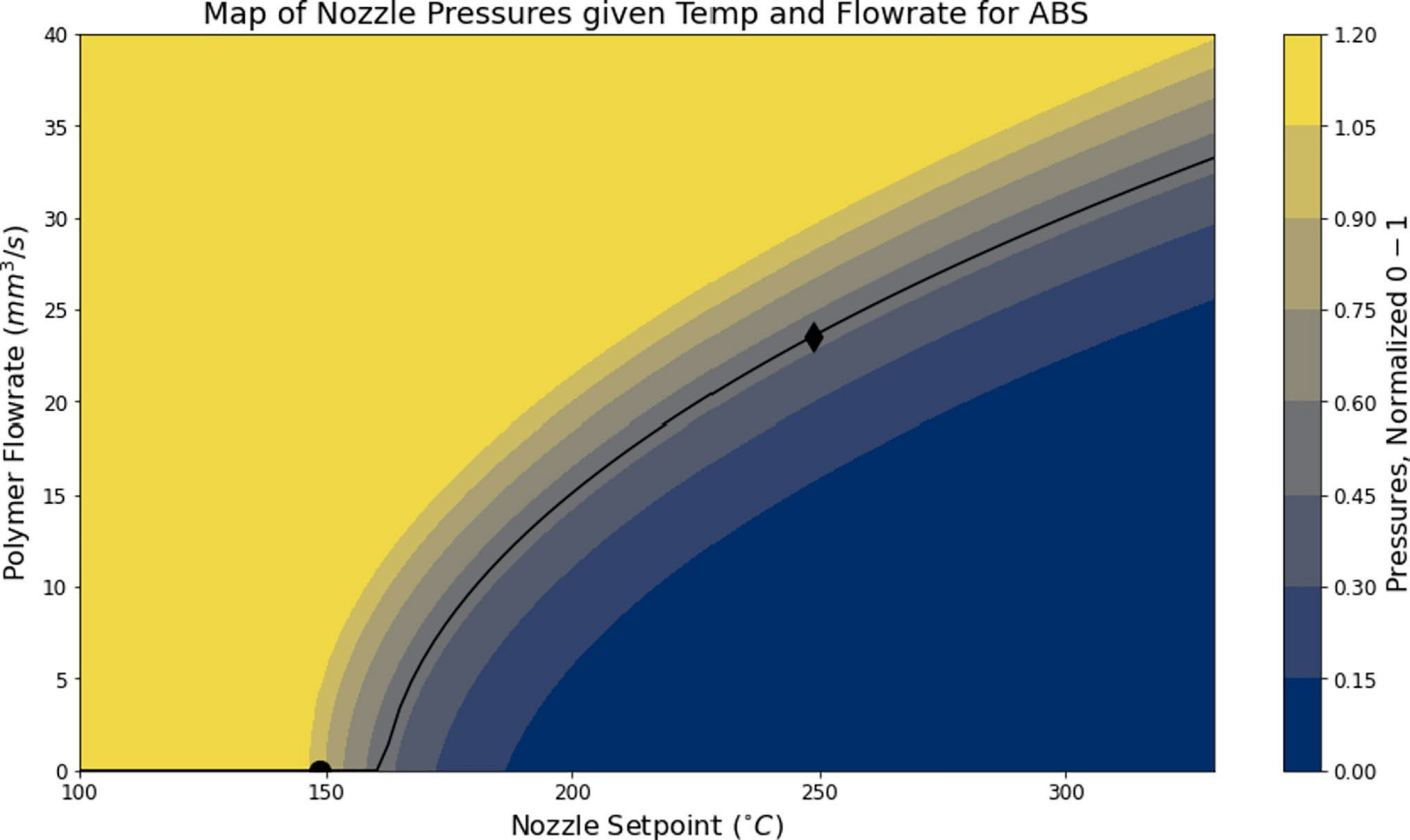
Here, we show a contour describing pressure *P* as a function of flow rate and temperature, as mapped to data from a 0.8mm nozzle in an E3D V6 Hotend using ABS filament. This fit matches parameters to Eq. (2): $c = 0.957, d = 65.2, e = -0.116, f = -154$. Here, we also show the temperature of first-flow (marked with a circle, around 150$^\circ \hbox {C}$) and our system’s selected maximal flow rate parameter (marked with a diamond, at 250$^\circ \hbox {C}$ and 23.5 mm$^3$/s) as described in “Extracting Real Parameters from Function Fits using Input Parameters”

We extended these fits for individual flow rates across the contour $P = f(T, Q)$ to map expected pressure as a function of any chosen operating temperature and flow rate. We observed that best-fit parameters for *b* were typically quadratic with respect to flow rate, and *a* parameters tangentially approached 1.0 with respect to flow rate, and developed Eq. 2 with parameters $c, d, e \ and \ f$ that we fit again using the same nonlinear least squares method. An example of one such fit is rendered in the plot in Fig. 4.

2 $$\begin{aligned} \begin{aligned} a&= \quad -c^{Q + d} + 1 \\ b&= \quad eQ^2 + f \\ P&= (-c^{Q + d} + 1)^{T + eQ^2 + f} \end{aligned} \end{aligned}$$

Interpretation of the *c*, *d*, *e*, and *f* parameters are better understood with relation to their *a* and *b* counterparts: for example, *f* maps to *b* at zero flow, meaning a temperature where flow is impossible even at near zero speeds (or more directly, where we would expect that measured pressure would equal 1.0, or the maximum pressure observed in the system prior to drive gear slip). The *e* parameter then indicates how quickly minimum temperatures increase with respect to flow rates. Parameters *c* and *d* seem less straightforward in their interpretability, given that the *a* from single fits is anyways fairly abstract.

### Extracting Real Parameters from Function Fits using Input Parameters

Our function fits are a useful underlying abstraction to describe expected nozzle pressures across a range of operating conditions, but they cannot tell us exactly what an optimal operating condition might be. For example, were we to suppose that print speed alone were optimal, our functions would tell us that printers should be operated near their maximum temperatures at all times—but existing practice shows this not to be the case. In order to deploy our function fits in available slicers and compare their outputs with existing heuristics, we deploy a set of input parameters that map between real-world and function fit locations.

The first parameter is $T_{rel}$, an offset in $^{\circ }C$ from the temperature identified in the function fit as the location where flow is first possible. For example, the function fit in Fig. 4 reports initial flow at 154$^\circ \hbox {C}$, meaning a $T_{rel} = {80}^\circ \hbox {C}$ would select $T = {234}^\circ \hbox {C}$; i.e., $T_{operating} = T_{firstFlow} + T_{rel}$. The second parameter is a relative pressure $P_{rel}$. It selects a flow rate at the provided temperature, by specifying desired nozzle pressure from $0 \rightarrow 1$, where 1 is the maximum flow possible before exceeding the extruder’s generative force.

We reasoned that, given our function fits as an underling abstraction, we could find one set of input parameters that would suit all machine configurations. To do so, we compared our function fits against heuristics for two common materials (PLA and ABS) with one highly common machine configuration (an E3D V6 Hotend with a 0.4mm Nozzle) and one rare configuration (the same hotend with a 0.8mm Nozzle). The results from that comparison are in Tables 4 and 5. The tables also references four varying flow rates, each of which is found within state-of-the-art slicers: an explicitly set **max** rate, and then tracks with **high, medium, and low** relative geometric importance (which are implicitly set). Based on this comparison, and using our own heuristic understanding of the process, we reasoned that we would select a $T_{rel} = {80}^\circ \hbox {C}$ and $P_{rel} = 0.75, 0.20, 0.10$ and 0.05 for maximum rates and low, medium and high track importance, respectively.

**Table 4:** Here, we tabulate heuristic nozzle set points against temperatures of first-flow from data gathered using our tool and function fit, to inform our choice of a stable input parameter for $T_{rel}$

Material, nozzle	Heuristic $(^{\circ }C)^1$	First flow $(^{\circ }C)^2$	Equivalent $T_{rel}\,(^{\circ }C)$
PLA 0.4	210	141.8	68.2
ABS 0.4	255	166.6	88.4
PLA 0.8	220	136.6	83.4
ABS 0.8	265	154.4	110.6

$^1$ Extracted from PrusaSlicer 2.5.0 using *Generic* polymer profiles

$^2$Using a 0.4mm Nozzle with an E3D V6 Hotend

**Table 5:** Here, we compare heuristic flow rates into pressures as defined by our data gather and fit, in order to inform our choice of stable input parameter for $P_{rel}$

Material, nozzle	Typical rate	Heuristic (mm$^3$/s)	Equivalent $P_{rel} \; (\%)$
PLA, 0.4$^4$	Max	15.0	0.681
	High $^1$	7.20	0.092
	Medium $^2$	4.05	0.044
	Low $^3$	2.25	0.030
ABS, 0.4	Max	11.0	0.098
	High	7.20	0.033
	Medium	4.05	0.013
	Low	2.25	0.007
PLA, 0.8$^5$	High	18.00	0.244
	Medium	12.60	0.074
	Low	9.00	0.026
ABS, 0.8	High	18.00	0.142
	Medium	12.60	0.044
	Low	9.00	0.018

$^1$External Perimeters, Small Perimeters, Bridges, Gap Fill

$^2$Perimeters, Top Solid Infill, Support Material, Support Interface

$^3$Infill and Solid Infill

$^4$For 0.4mm nozzles, we calculated flow rates using 0.2mm track heights and 0.45mm track widths, which are defaults in the PrusaSlicer “Quality” print configurations

$^5$For 0.8mm nozzles, we calculated flow rates using 0.4mm track heights and 0.90mm widths, which are defaults in the PrusaSlicer “Quality” print configurations

**Table 6:** The input parameters that we chose to use in our deployment of our system in the evaluation/results section of this paper

Material, nozzle	Typical rate	Selected $T_{rel} (^{\circ }C)$	Selected $P_{rel}$
Any, any	Max	80	0.750
	High	80	0.250
	Medium	80	0.100
	Low	80	0.050

### Deploying Flow-Based Parameters in Conventional Slicers

In order to complete our experiment, we finally need to convert our chosen parameters (which are described in terms of polymer flows) into parameters that can be interpreted by off-the-shelf slicers (where flow rates are implicit). First, our method chooses flow rates for four types of printer instructions: Maximal, and then high, medium, and low rates as shown in Tables 5 and 6. These categories match to groups of parameters we found in PrusaSlicer, for example maximal speeds are typically used during printer infill, high speeds for top and bottom solid infills, medium speeds on external perimeters and low speeds on small perimeters. In general, most heuristically developed parameter sets tend to assign lower speeds to finer detailed geometries, and higher speeds to invisible or bulky parts of a print (like infill).

Once our method calculates flow rates in mm$^3$/s for these four speed categories, it outputs equivalent linear feed rates in mm/s for a selected track width and layer height. We then manually input these selections into the slicer in order to generate GCode and print the test artifacts. This obvious shortcoming of the method is a primary focus of our future work, as we discuss in section “Integrating with Motion Control and Slicing.”

### System Summary

**Fig. 5:**
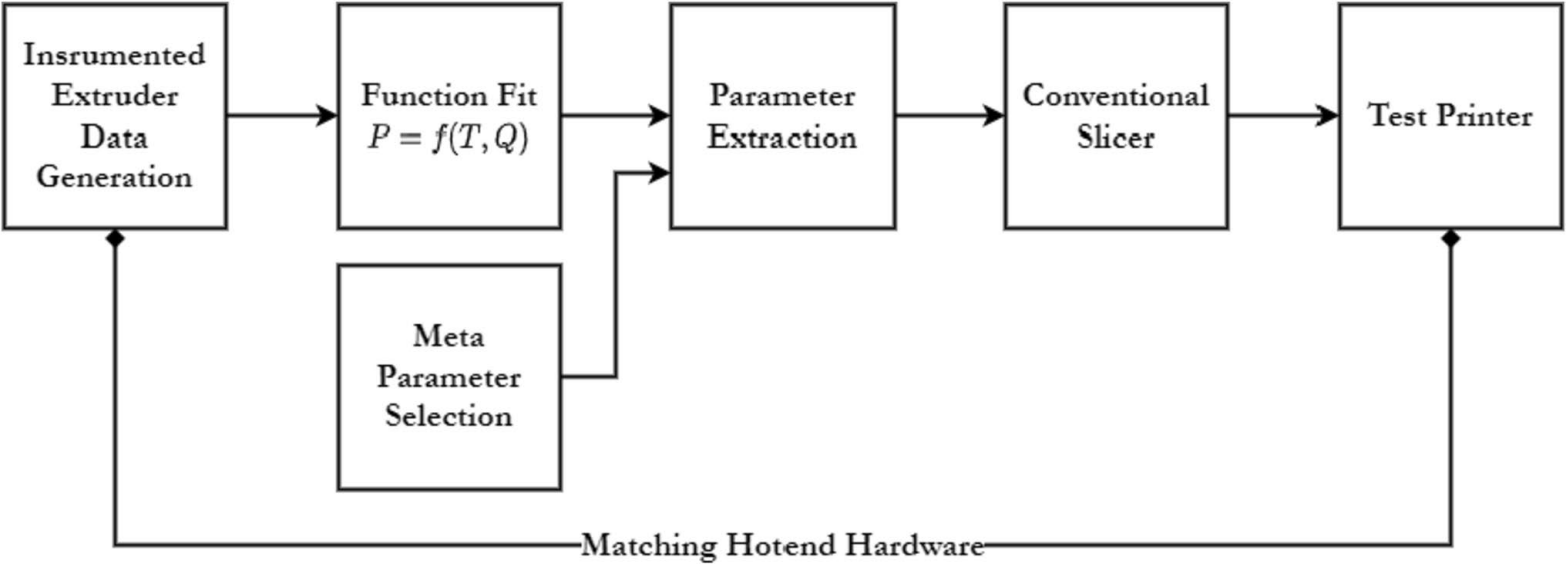
In our evaluation of this method to automatically select print parameters, we deploy the function fit and test data on test prints, by matching a test printers’ hotend configuration to that of the instrumented extruder and running extracted parameters through an off-the-shelf slicer

Our end-to-end method for the automatic selection of print parameters is complete in five steps, which we diagram in Fig. 5. First, we use our instrumented extruder (outfit with the same hotend hardware as our test printer) to generate a dataset. That dataset is fit against the described function, and that function fit is used to extract real parameters using our chosen input parameters. To show the viability of this method for extending heuristics across multiple materials and nozzles, we used the same input parameters in each print shown in the evaluations section; those parameters are rendered in Table 6. Extracted parameters are then processed using an off-the-shelf slicer (we used PrusaSlicer 2.5.0), as described in section “Deploying Flow-Based Parameters in Conventional Slicers” and test instructions are sent to a test printer (a Prusa MK3).

## Results

**Fig. 6:**
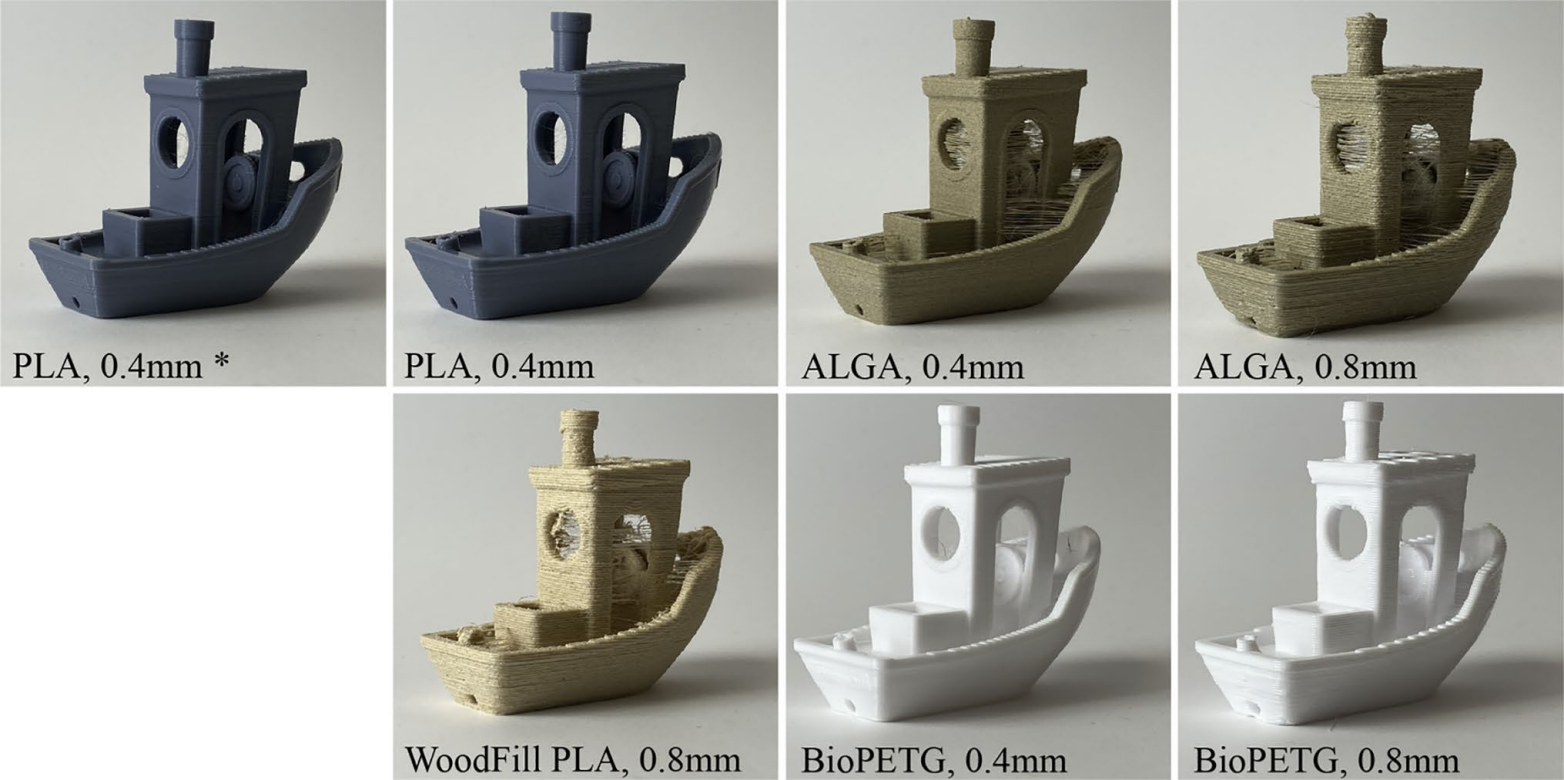
We deployed our process to produce print parameters for six unique machine, material configurations. We then used those parameters to print the *3DBenchy* model [35], a common benchmarking artifact among FFF users and researchers. We include here, one model printed using heuristically available parameters, which is marked with an asterisk. Our method was able to produce viable print parameters for each filament we tested

We printed the *3DBenchy* model using parameters generated with our method in order to demonstrate its viability. In Fig. 6, we include images of the resulting prints, and in Table 7 we include the temperature and flow rate parameters that the method produced, including (for reference) the heuristic data that were available to us once we had purchased these filaments.

Our method produced temperature selections that were within the manufacturer’s specification in all but one case, and was able to automatically produce viable flow rate parameters where none were otherwise available. None of the prints resulted in failures of any kind, although stringing was visible in two of the four filaments tested.

**Table 7:** Here, we include the parameters generated by our process during our evaluation, matching the images of printed artifacts from Fig. 6

Configuration	Heuristic			Our Method		
	Temp	Flows	Time	Temp	Flows	Time
	$^\circ \hbox {C}$	mm$^{3}$/s	min	$^\circ$C	mm$^{3}$/s	Min
Generic PLA 0.4	210	7.20, 4.05, 2.25	89	222	12.71, 9.39, 6.84	78
Woodfill PLA 0.4$^1$	190–210	Not provided	n/a	223	12.92, 9.64, 7.12	33
ALGA 0.4	185–210	Not provided	n/a	206	15.63, 11.81, 8.96	79
ALGA 0.8	185–210	Not provided	n/a	203	19.37, 14.86, 12.1	33
Bio PETG 0.4	225–230	Not provided	n/a	236	13.95, 7.47, 1.69	94
Bio PETG 0.8	225–230	Not provided	n/a	216	15.35, 8.11, 2.75	53

$$^1$$40% Wood

## Limitations and Future Work

The basic premise in this work is that FFF print parameters should be based mostly on FFF phenomenology; namely nozzle temperatures and flow rates. We reasoned that, if we were able to characterize just this process using a simple abstraction, we could make improvements to the way print parameters are selected, using data as a basis for parameter selection rather than simply trialing heuristic selections. While our process does work fairly well, it has become clear to us that nozzle phenomenology alone is not enough to select parameters.

### Extracting Thermodynamic Models from Data Traces

In section “Fitting Individual Flow Rates” we noted that the *a* parameter is likely related to nozzle thermodynamics, observing that lower flow rates correspond to more pronounced pressure drop off with respect to temperatures (more complete heat transfer) and higher flow rates to more “stubborn” pressure traces. We suspect that, at higher rates, the filament simply does not spend enough time in the melt zone to come up to the nozzle’s set point temperature.

**Fig. 7:**
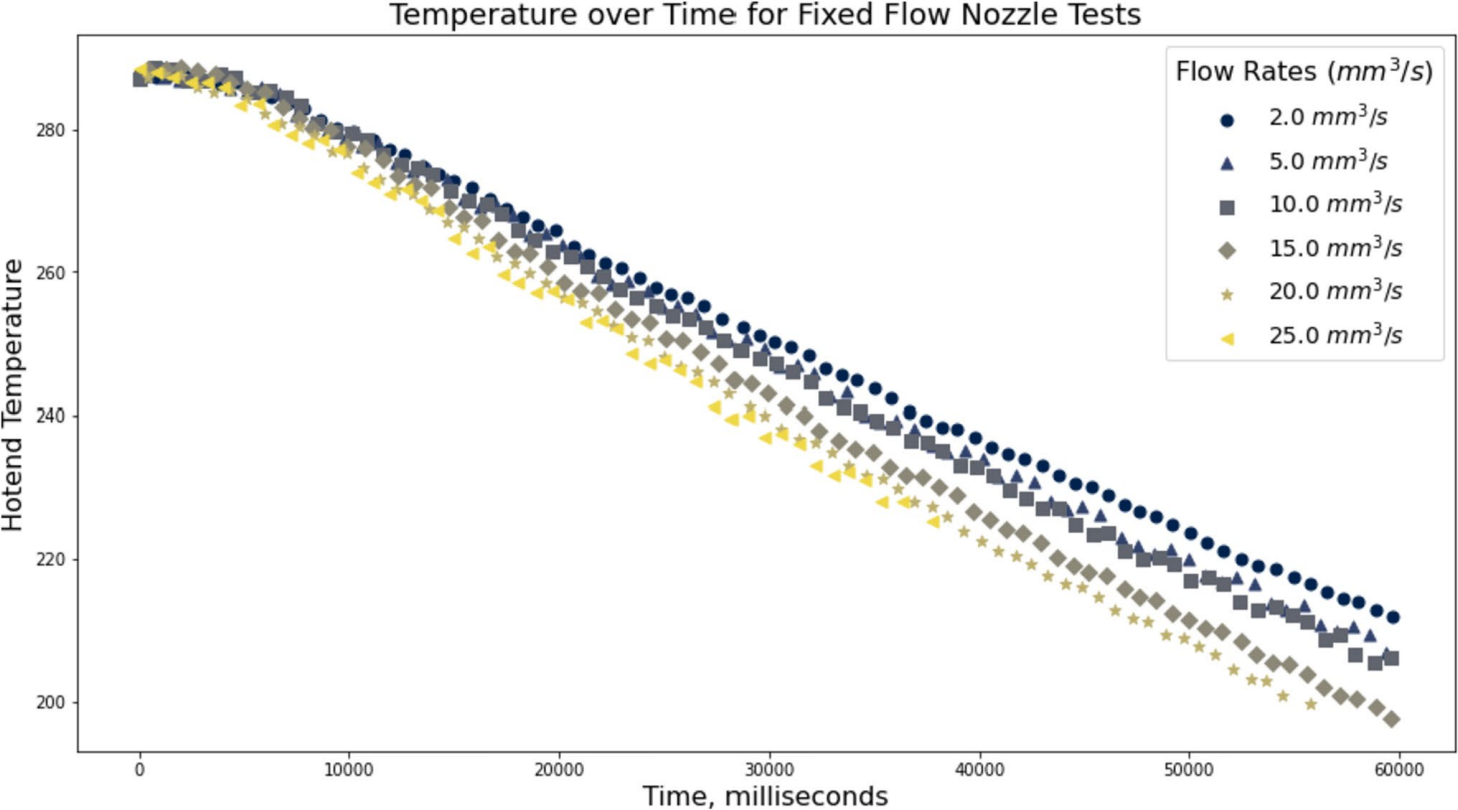
Here, we show the potential of capturing thermodynamic data from the data gathering procedure discussed in section “Data Gathering and Normalization.” This plot renders hotend temperature over time, and shows that increases in flow rate result in faster drops in temperature due to increases in the heat transfer into the melt flow. We hope that, in future work, we can extract simple thermodynamic models of a machine’s hotend using the same type of data

These thermodynamics are a key limit to FFF printing, as studied extensively in [27]. Optimal control of an FFF machine should include a thermodynamic model of the hotend that could explain the phenomenology we observe in our data, and it seems likely to us that extracting such a model from the datasets generated here, is possible. To illustrate the presence of calorimetric data here, we include Fig. 7 that renders the same data from Fig. 7 but re-organized to show how different flow rates correspond to varying rates of hotend cooling.

### Integrating with Motion Control and Slicing

A core limit to our method is that the slicing process itself is entirely disconnected from actual real-time control of FFF systems. Because motion controllers apply trajectory optimizations on top of selected parameters, flow rates that we select are sometimes not actually achieved during machine operation. This is a broader limit to the advancement of the FFF process that is discussed in more detail in [11]. A similar limit is present to researchers of five-axis machining toolpaths [36].

This is clear also when we look at our results for six *3DBenchy* prints in Table 7; while our method selects flow rates that are roughly twice that of the heuristic selections, the Benchy part is only produced 12% faster overall. This is indicative that the machine’s overall rate was more constrained by acceleration limits than by flow rate limits.

Combining motion control optimizations with FFF-specific optimizations on flow rates and temperatures is a logical next step, and we are also developing a modular, software-based motion controller code to do so [37].

Slicers also express parameters in a manner that is fundamentally incompatible with this method: most use linear feed rates to describe print settings, even though it has become widely acknowledged that polymer flow rate is the major limiting factor in FFF processing speeds. COTS slicers’ organization around linear feed rates is not without warrant; higher speeds typically correlate negatively with print quality simply due to limits in a machine’s motion system. We hope that the authors of the next generation FFF slicers will be able to strike some compromise in expressing both of these coupled limits to process tuners, and that this work can contribute to that discussion an idea about dimensionality reduction in parameter selections—that is, reducing complex and exhaustive parameter sets into more concise and expressive models.

### Comparing Blind vs Model-Informed Search

In this paper we present a simple function fitting approach to capture machine phenomenology. It works well in this small experiment, but more complex systems may warrant other approaches. Future work will involve comparing strategies with few or no priors (blind search) against model-informed search to explore the trade-offs between data requirements vs. modeling complexity.

### Evaluation Methods

We acknowledge that this paper itself carries out a limited evaluation of the method, using only qualitative analysis of print quality and a simple quantitative printing speed metric. An improved study could implement a more rigorous geometric analysis of printed parts for accuracy, as well as layer adhesion and part strength tests.

## Conclusion

While this work does not make a complete reckoning with all of the phenomenology and modeling associated with FFF printing that may be required in order to select *optimal* parameters, it does show that even simple methods in combination with instrumented hardware and workflows that connect machines to slicers can have promising results.

We showed that a small dataset, generated quickly using online FFF instrumentation, can be enough to automatically select print parameters for otherwise unknown machine configurations.

The method holds particular relevance for individuals involved in slicer authorship, machine design, and related domains as it provides an alternative to the exhaustive and labor-intensive process of hand-tuning parameter sets. We hope that the work will contribute to the ongoing proliferation of FFF, the adoption of more novel machine designs and filament selections, and an increased ubiquity of making in the world.

## Footnotes

1 Certain commercial equipment, instruments, or materials are identified in this paper in order to specify the experimental procedure adequately. Such identification is not intended to imply recommendation or endorsement by the National Institute of Standards and Technology, nor is it intended to imply that the materials or equipment identified are necessarily the best available for the purpose.
